# Effect of Muscle Contraction Under Caloric Restriction on Irisin and FGF21 Secretion in Mice

**DOI:** 10.33549/physiolres.935581

**Published:** 2025-12-01

**Authors:** Riku TANIMURA, Hiroyuki WATANABE, Takanaga SHIRAI, Kazuki UEMICHI, Tomohiro IWATA, Tohru TAKEMASA

**Affiliations:** 1 Graduate School of Comprehensive Human Sciences, University of Tsukuba, Tsukuba, Japan; 2 Japan society for the promotion of science, Tokyo, Japan; 3 Department of Human Sciences, Kanagawa University, Kanagawa, Japan; 4 Institute of Health and Sports Sciences, University of Tsukuba, Tsukuba, Japan

**Keywords:** Muscle, Calorie restriction, Myokine, Irisin, FGF21

## Abstract

Combining diet and physical activity is known to be more effective for health than either intervention alone. Recent research has shown that skeletal muscle secretes myokines in response to exercise, which contribute to the adaptation of other organs to exercise. Therefore, we hypothesized that muscle adaptation by calorie restriction (CR) might enhance myokine responses to exercise. It is known that the myokine fibroblast growth factor-21 (FGF-21) secreted by skeletal muscle during exercise activates adipose tissue browning. We have already reported that irisin, a myokine that contributes to the metabolic activation of adipose tissue and weight loss, is secreted in response to muscle contraction by electrical stimulation (ES). Thus, we investigated the secretion of FGF21 and irisin upon the combination of ES with CR in this study. Mice were divided into four groups: control mice (Con), calorie restriction mice (CR), acute muscle contraction mice (ES), and acute muscle contraction after calorie restriction mice (CRES). After 1 week of acclimation, we subjected the mice to 60 % calorie restriction. After 2 weeks of CR, we performed ES. The results showed that the irisin expression level in serum was significantly increased by the combination of ES and CR, and an interaction between CR and ES was confirmed. FGF21 expression in serum was significantly decreased by CR. In conclusion, we confirm that irisin is a myokine whose secretion is increased synergistically by CR and muscle contraction.

## Introduction

Health problems caused by lifestyle-related diseases such as obesity and type 2 diabetes are becoming a huge social issue. Exercise and dietary interventions have thus attracted attention as inexpensive and effective countermeasures to overcome these issues. In a previous study on exercise, endurance training or high-intensity interval training significantly prevented the increases in body weight and total body fat mass caused by a high-fat diet in mice [1]. Another study showed that 8 weeks of endurance training improved insulin sensitivity and reduced inflammation in obese mice [2]. We have also reported that 4 weeks of endurance training reduced adipocyte size and stimulated thermogenesis of inguinal white adipose tissue in mice [3,4]. Moreover, in a previous study in humans, a training program lasting 12 weeks comprising resistance or aerobic exercise, or them in combination, improved the cardiovascular risk profile in overweight subjects, compared with the status after performing no exercise [5]. As mentioned above, exercise has been reported to improve overall human health and wellness. Many health benefits of dietary interventions have also been reported. In particular, calorie restriction (CR) is widely used to boost longevity and prevent lifestyle-related diseases [6]. CR not only reduces body weight and fat mass but also decreases the incidence of aging-related diseases such as cardiovascular disease and diabetes [7,8]. In rodent studies, it has similarly been reported that CR improves insulin sensitivity and the thermogenic capacity of adipose tissue [9,10]. Given that each of exercise and CR has beneficial effects on the body, their performance in combination may have a synergistic effect. Indeed, in a previous study, it was found that the combination of CR and endurance exercise synergistically suppressed high-fat diet-induced obesity and insulin resistance [11]. Another study indicated that the combination of CR and exercise exerted greater cardioprotective effects than either intervention alone by improving the cardiometabolic status in obese/insulin-resistant rats [12]. Although several studies have reported that CR and exercise exert synergistic effects on adipose tissue, the critical molecular mechanism behind these effects remains unclear. Therefore, we focused on the response of anti-obesity myokines to the combination of CR and exercise as factors potentially involved in their synergistic effects.

Myokines are proteins secreted by muscles that are known to be involved in the beneficial effects of exercise [7]. They are secreted in response to muscle contractions, certain nutritional conditions, and hormonal stimulations [13]. In particular, myokines induced by exercise have been reported to improve physiological adaptation to exercise. For example, fibroblast growth factor-21 (FGF-21) secreted by skeletal muscles during exercise directly regulates processes of bone repair [14,15]. It has also been reported that FGF-21 regulates peroxisome proliferator-activated receptor gamma coactivator 1-α (PGC-1α) and the browning of white adipose tissues in adaptive thermogenesis [16]. In addition, irisin, which is cleaved from Fibronectin Type III Domain Containing 5 (FNDC5), a membrane protein of myotubes, and then secreted, promotes the browning of white adipose tissue [17]. Against this background, the skeletal muscle is considered to be not only a motor organ but also a secretory one. Meanwhile, much of the previous research on the responses of myokines to contractions has been conducted on skeletal muscle under normal dietary conditions. Since skeletal muscle exhibits plasticity in its response to nutritional status, there is a need to confirm the effect on muscle adaptation of the myokine response to muscle contraction under CR. Any myokines showing synergistic increases in their secretion upon the contraction of mouse skeletal muscle under CR are likely to be factors mediating the synergistic health-promoting effects of exercise and CR.

In this study, we investigated the effects of CR on the myokine response to muscle contractions. We induced muscle contractions in mice via electrical stimulation (ES). This approach is known to induce high-intensity muscle contractions. In this model, contractions of the gastrocnemius muscle are induced by transcutaneous ES using electrode pads attached to the lower leg. In our previous study, we demonstrated that electrical stimulation (ES) activates downstream signaling of mTOR in skeletal muscle and promotes muscle protein synthesis [18,19]. In addition, we previously reported that acute ES induced an increase in irisin expression in lower leg muscle and serum [20]. We considered electrical stimulation (ES) to be an appropriate approach for analyzing myokines triggered specifically by muscle contraction and therefore adopted this method in our study. We provided mice in the CR group with 60 % of the mean amount of food that they consumed for 2 weeks. Our previous studies suggested that 2 weeks of 60 % CR is sufficient to promote adaptations in muscle quality and quantity [21,22]. We performed acute ES after 2 weeks of CR and evaluated myokine secretion.

## Materials and Method

### Experimental approval

Animal experiments were conducted humanely under approval from the Institutional Animal Experiment Committee of the University of Tsukuba (approval number 23-397), in accordance with the university’s Regulations for Animal Experimentation and the Fundamental Guidelines for Proper Conduct of Animal Experiments and Related Activities in Academic Research Institutions under the jurisdiction of the Ministry of Education, Culture, Sports, Science and Technology, Japan.

### Animals

Male CD^®^-1 HaM/ICR mice aged 7 weeks (Charles River Laboratories Japan, Kanagawa, Japan) were used in this study. The mice were housed in temperature (22 °C ± 2 °C) and humidity (55 % ± 5 %)-controlled holding facilities under a 12/12 h light/dark cycle and were provided with ad libitum access to water. The mice were divided into four groups: sedentary group (CON; n = 6), muscle contraction group (ES; n = 6), sedentary and CR group (CR; n = 6), and muscle contraction and CR group (CRES; n = 5). The sample size was smaller in the CRES group because one of the animals died due to anesthesia at the time of ES. Consistent with our previous study [22], the food intake of the CR group was set at 60 % of that of the AL group. Before intervention, the average amount of food consumed by each mouse was calculated based on daily intake measurements during the one-week of acclimation. During the intervention, each mouse in the CR group was provided with 60 % of its own average food intake determined during acclimation. Fourteen days after the experiments were completed, the mice in the ES group underwent ES. Blood was collected immediately thereafter, and serum was extracted from the blood. After blood collection, the muscles were excised, weighed, quickly frozen in liquid nitrogen, and stored at −80 °C until further analyses. Based on previous studies indicating that the secretion of many exercise-induced myokines peaks immediately after exercise, tissue and blood samples were collected immediately following ES [23,24].

### Muscle contraction protocol

The muscle contraction protocol was carried out as described previously [18,20,25]. Briefly, under inhaled isoflurane anesthesia (2 %, KN-1701; Natsume), the lower legs of each mouse were shaved and cleaned with alcohol wipes. The mouse foot was positioned on a footplate (with an ankle joint angle of 90°) in a prone position. The triceps calf muscle was isometrically exercised (10 × 3-s stimulation, with 7-s interval between contractions: total of five sets with 3-min interval between sets/a total duration of approximately 20 minutes). The voltage (30 V) and stimulation frequency (100 Hz) were adjusted to produce maximal isometric tension with electrodes connected to an electric stimulator and isolator (Ag/AgCl, Vitrode V; Nihon Kohden). For the control animals, anesthesia and shaving of the lower legs were performed for the same duration as in the ES and CRES groups. Previous study demonstrated that this exercise protocol is known to increase anabolic signaling activity [18,19,26] and induces significant muscle hypertrophy, simulating long-term training [27].

### Western blotting

Total muscle proteins were extracted using lysis buffer containing 50 mM HEPES (pH 7.6), 150 mM NaCl, 10 mM EDTA, 10 mM Na_4_P_2_O_7_, 10 mM NaF, 2 mM Na_3_VO_4_, 1 %(vol/vol) NP-40, 1 %(vol/vol) Na-deoxycholate, 0.2 %(wt/vol) SDS, and 1 %(vol/vol) of a complete protease inhibitor cocktail (Nacalai Tesque Inc.). Concentrations of muscle proteins were measured using a Protein Assay Bicinchoninate Kit (Nacalai Tesque Inc.). Just before sodium dodecyl sulfate-polyacrylamide gel electrophoresis (SDS-PAGE), an aliquot of the extracted muscle protein solution was mixed with an equal volume of sample loading buffer containing 1 % (vol/vol) 2-mercaptoethanol, 4 % (wt/vol) SDS, 125 mM Tris–HCl (pH 6.8), 10 % (wt/vol) sucrose, and 0.01 %(wt/vol) bromophenol blue, and serum was mixed with loading buffer to contain 5 % of each sample. The mixture was then heated at 37 °C for 1 h. Ten micrograms of the protein mixtures were separated on an SDS-PAGE gel and then electrically transferred from the gel to a polyvinylidene difluoride membrane (Bio-Rad Laboratories). The membrane was blocked using 50 % Blocking One (Nacalai Tesque) in Tris-buffered saline with 0.1 % Tween-20 (TBST) for 1 h at room temperature and incubated with primary antibodies overnight at 4 °C in Can Get Signal Solution 1 (TOYOBO). After treatment with the primary antibody, the membrane was washed three times for 10 min with TBST and incubated with secondary antibodies for 1 h at room temperature in Can Get Signal Solution 2 (TOYOBO). After washing with TBST three times for 15 min each, the signals were detected using ImmunoStar Zeta or LD (Wako Chemicals), quantified by C-Digit (LI-COR Biosciences), and expressed as arbitrary units. Ponceau staining was used to confirm the consistency of the loading. The following primary antibodies were used for western blotting: anti- phospho-rpS6 (Ser240-244) (4858S; Cell Signaling Technology), anti-S6 Ribosomal protein (2217; Cell Signaling Technology), anti-AMP-activated protein kinase (AMPK, 2532S; Cell Signaling Technology), anti-phosphorylated AMPK (2535S; Cell Signaling Technology), anti-extracellular signal-regulated kinase (ERK1/2, 9102; Cell Signaling Technology), anti-phosphorylated ERK1/2 Thr202/Tyr204 (4370; Cell Signaling Technology), anti- PGC-1α (516557; Millipore), anti-FNDC5/irisin (ab174833; Abcam), and FGF21 (ab171941; Abcam).

### RNA isolation and real-time polymerase chain reaction (PCR)

Total RNA (mRNA) was isolated from frozen whole gastrocnemius muscles using TRI reagent (Invitrogen). The quantity and quality of RNA were validated with NanoDrop (Thermo Fisher Scientific). Complementary DNA was synthesized using the PrimeScript RT Master Mix (Takara Bio, Inc.). qRT-PCR was performed with the Thermal Cycler Dice Real-Time System using SYBR Premix Ex Taq II (Takara Bio, Inc.). The PCR protocol was as follows: denaturation for 15 s at 95 °C, followed by annealing and extension for 60 s at 60 °C (40 cycles). The dissociation curve for each sample was analyzed to verify the specificity of each reaction. Relative mRNA expression levels were determined using the standard curve method and normalized three housekeeping genes (HKGs): hypoxanthine phosphor-ribosyl transferase (*Hprt*), TATA-box binding protein (*Tbp*), and *β-actin*. The primer sequences are shown in Table 1.

### Statistical analysis

Data are presented as mean with individual data points. GraphPad Prism 10 software (GraphPad, Inc.) was used for all statistical calculations. The significance of differences was analyzed by two-way analysis of variance (ANOVA). In the case of significant F values, comparisons were performed using Bonferroni post hoc test. The significance level was set to *p* < 0.05.

## Results

### Animal characteristics

**Fig. 1:**
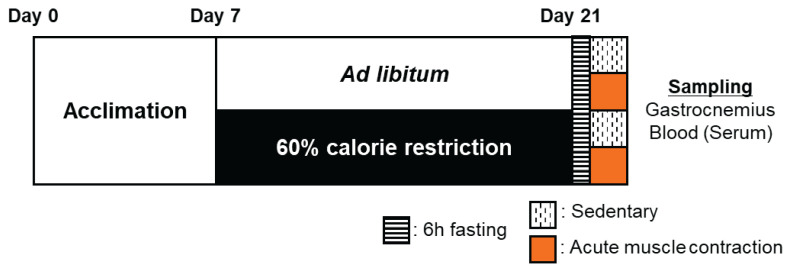
Experimental design. Mice were divided into four groups: control mice (Con), calorie restriction mice (CR), acute muscle contraction mice (ES), and acute muscle contraction after calorie restriction mice (CRES). After 1 week of acclimation, we performed calorie restriction. After 2 weeks of CR, we performed ES. n = 5–6 per group.

**Fig. 2:**
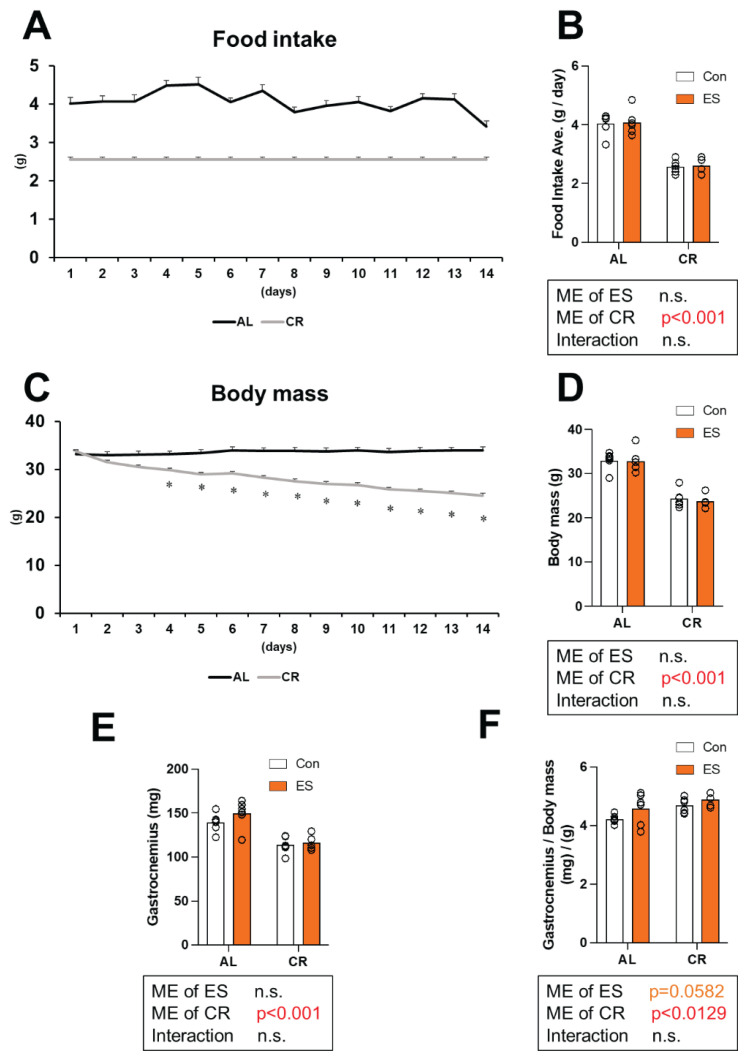
Effect of acute muscle contraction by electrical stimulation (ES) and calorie restriction (CR) on food intake and phenotype of mice. (**A**) Changes in food intake, (**B**) mean values of food intake over the entire period, (**C**) changes in body mass, (**D**) mean values of body weight at the time of dissection, (**E**) gastrocnemius wet weight, and (**F**) gastrocnemius wet weight per body mass. Data are presented as mean with individual data points (n = 5–6). The significance of differences was analyzed by two-way analysis of variance (ANOVA). The significance level was set to *p* < 0.05. The p-values for the main effects (ME) of CR and ES, as well as their interaction, are indicated below each histograms.

The protocols of this study are shown in Fig. 1. This study was conducted with four groups of mice, but the only intervention performed until day 14 was CR, so the changes in food intake and body weight until 14 days are shown in the graphs for the two groups (AL vs. CR). A significant main effect of CR was observed for food intake (p < 0.001) (Fig. 2A, 2B). A significant main effect of CR on body weight was found from day 3 of the experiment (p<0.001) (Fig. 2C). A significant main effect of CR was observed on body mass on day 14 (p<0.001) (Fig. 2D). A significant main effect of CR was observed on the wet weight of the gastrocnemius muscle (p<0.001) (Fig. 2E). Meanwhile, a significant main effect of CR was also found for the ratio of gastrocnemius muscle weight to total body weight (p = 0.0129), and a non-significant trend was observed for ES (p=0.0582) (Fig. 2F). These results showed that 2 weeks of CR induced weight loss and muscle atrophy.

### Protein expression in skeletal muscle

To provide evidence supporting the ES-induced muscle contraction, we measured representative signaling pathways altered by ES, including rpS6, AMPK, and

**Fig. 3:**
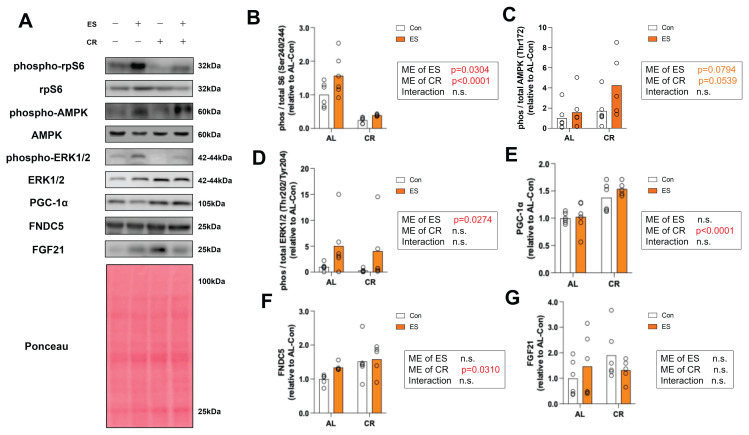
Effect of acute muscle contraction by electrical stimulations (ES) and calorie restriction (CR) on protein expression levels in gastrocnemius. (**A**) Representative immunoblots. (**B**) Relative phosphorylation level of rp-S6, (**C**) Relative phosphorylation level of AMP-kinase (AMPK), (**D**) Relative phosphorylation level of Extracellular signal-regulated kinase (ERK1/2), (**E**) Relative expression level of Peroxisome proliferator-activated receptor gamma coactivator 1-α (PGC-1α), (**F**) Relative expression level of Fibronectin type III domain-containing protein 5 (FNDC5), (**G**) Relative expression level of Fibroblast growth factor 21 (FGF21) analyzed by western blotting. Data are presented as mean with individual data points (n = 5–6). The significance of differences was analyzed by two-way analysis of variance (ANOVA). The significance level was set to p < 0.05. The p-values for the main effects (ME) of CR and ES, as well as their interaction, are indicated beside each histograms.

ERK1/2. A significant main effect of ES (p=0.0304) and a significant main effect of CR (p<0.0001) were observed on rp-S6 phosphorylation (Fig. 3B). No significant main effects were found for AMPK phosphorylation, although a tendency of CR (p=0.0794) and ES (p=0.0539) was noted (Fig. 3C). A significant main effect of ES was observed for the phosphorylation level of ERK1/2 (p=0.0274) (Fig. 3D). We also examined the expression level of PGC-1α, transcriptional regulator of FNDC5, and FNDC5, a precursor protein of irisin, as findings have suggested its potential involvement in the secretion of myokines into the blood. A significant main effect of CR was observed on the expression of PGC-1α (p<0.0001) (Fig. 3E). A significant main effect of CR was observed on the expression of FNDC5 (p=0.0310) (Fig. 3E). No significant differences in FGF21 protein expression levels were observed between the groups (Fig. 3G). Therefore, ES activated a molecular signal triggered by muscle contraction in the gastrocnemius muscle. We also found that 2 weeks of CR increased the expression of FNDC5 in gastrocnemius muscle, but FGF21 did not.

### Myokine mRNA expression in skeletal muscle

**Fig. 4:**
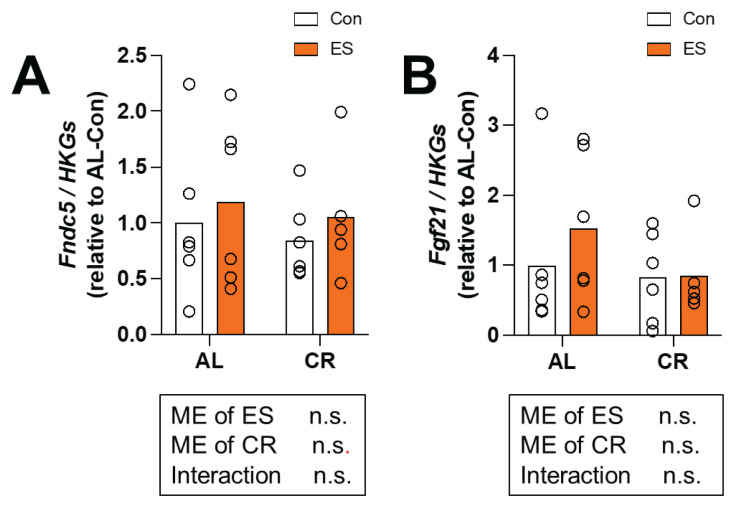
Effect of acute muscle contraction by electrical stimulations (ES) and calorie restriction (CR) on mRNA levels of myokines in gastrocnemius muscle. (**A**) Relative gene expression level of Fibronectin type III domain-containing protein 5 (*Fndc5*) and (**B**) Relative gene expression level of Fibroblast growth factor 21 (FGF21) analyzed by RT-PCR. Each value was corrected using the mean expression of three housekeeping genes (*Hprt*, *Tbp*, and *β-actin*). Data are presented as mean with individual data points (n = 5–6). The significance of differences was analyzed by two-way analysis of variance (ANOVA). In the case of significant F values, comparisons were performed using Bonferroni post hoc test. The significance level was set to *p* < 0.05. The p-values for the main effects (ME) of CR and ES, as well as their interaction, are indicated below each histograms.

We examined the mRNA expression levels of myokines in skeletal muscle. First, we analyzed *Fndc5*, a precursor protein of irisin. Unexpectedly, *Fndc5* did not exhibit any significant differences among all groups (Fig. 4A). Next, we examined the gene expression levels of *Fgf21*, but no significant changes were observed in response to the interventions (Fig. 4B).

### Myokine protein expression in serum

**Fig. 5:**
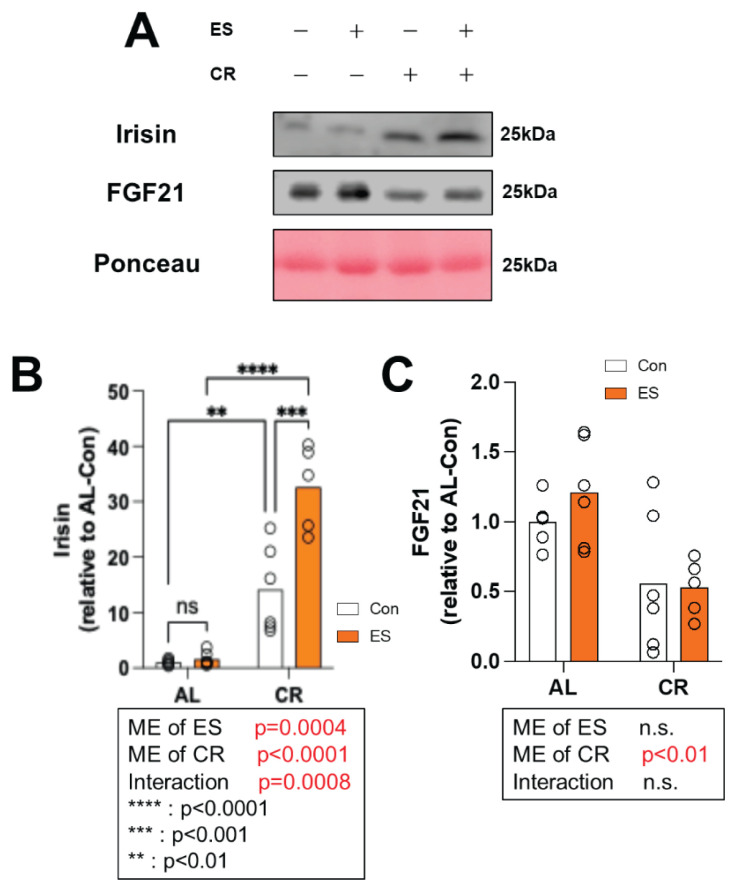
Effect of acute muscle contraction by electrical stimulations (ES) and calorie restriction (CR) on myokines in serum. (**A**) Representative immunoblots. (**B**) Relative expression level of Irisin and (**C**) Relative expression level of Fibroblast growth factor 21 (FGF21) analyzed by western blotting. Data are presented as mean with individual data points (n = 5–6). The significance of differences was analyzed by two-way analysis of variance (ANOVA). In the case of significant F values, comparisons were performed using Bonferroni post hoc test. The significance level was set to *p* < 0.05. The p-values for the main effects (ME) of CR and ES, as well as their interaction, are indicated below each histograms.

We examined the levels of myokine expression in the blood immediately after ES to characterize the secretion of myokines into the blood induced by muscle contraction. We examined the secretion of irisin and FGF21, which are known as browning factors. A significant main effect of ES (p=0.0004) and CR (p<0.0001) was observed on irisin protein levels in serum, and a significant interaction between CR and ES was found (Fig. 5B). A significant main effect of CR was observed for FGF21 protein levels in serum (p<0.01) (Fig. 5C).

## Discussion

In this study, we examined the myokine response to muscle contraction in mice subjected to CR. We induced acute high-intensity muscle contractions in mice after 2 weeks of CR. Our findings suggest that CR enhances irisin secretion in response to muscle contractions.

First, we confirmed the phenotypic adaptation of mice upon 2 weeks of CR. As in previous studies, body mass and muscle wet weight were decreased by 2 weeks of CR [21,22]. Meanwhile, the ratio of muscle wet weight to total body weight was significantly increased by CR and it showed a tendency to be increased by ES. Although we did not measure adipose tissue weight, these results suggest that fat mass loss rather than lean body mass loss contributes more to the weight loss caused by 2 weeks of CR. The increase of muscle wet weight by ES is consistent with the results of a previous study in which acute muscle contraction with blood flow restriction was found to increase muscle wet weight [28]. This is expected to be caused by a decrease in intracellular pH due to muscle contraction and the influx of blood to counteract it. Therefore, the samples obtained in this study reflected systemic adaptation by CR and acute response by ES.

Second, rp-S6 phosphorylation was increased by ES. This result is consistent with previous studies that served as references for the ES protocol used in this study[19,29]. AMPK phosphorylation was increased by ES (albeit not significantly). This is in line with our previous study that showed that AMPK phosphorylation was significantly increased in mouse skeletal muscle immediately after ES [20]. AMPK is a master regulator of exercise that senses the status of energy expenditure, promotes metabolism for glucose and fatty acid utilization, and mediates beneficial cellular adaptations in many vital tissues and organs [30]. Thus, ES partially reproduced the molecular response of skeletal muscle to exercise. In addition, ERK1/2 phosphorylation, which is responsive to mechanical stimulation, was increased by ES [31]. These findings suggest that ES stimulated muscle to contract and activated an intracellular molecular signaling cascade. Our results also suggest that CR increased PGC-1α and FNDC5 expression in mouse skeletal muscle. A previous study reported that 8 months of exposure to a CR diet increased the expression of FNDC5 in mouse skeletal muscle compared with AL [32]. Another study reported that the administration of AICAR, an adenosine analog and AMPK activator, enhanced FNDC5 expression in C2C12 cells [33]. Therefore, the increase of FNDC5 expression in mouse skeletal muscle by 2 weeks of CR could have been due to activation of the AMPK/PGC-1α signaling pathway. We next measured the mRNA expression levels of myokines in skeletal muscle. RT-PCR revealed discordant results regarding the mRNA and protein expression levels of FNDC5. In previous studies, a 48-h fast decreased the mRNA expression of FNDC5 in muscle [34], while CR for 8 months increased the expression of FNDC5 protein in skeletal muscle [35]. Considering these findings, we reconfirm that CR increases FNDC5 protein expression in skeletal muscle; it is also possible that the mRNA results in this study were masked by the period of fasting prior to sampling. It has also been reported that spatial factors and post-transcriptional regulation can lead to differences between mRNA and protein expression levels [36,37]. Therefore, these mechanisms should be considered as potential causes of the observed discrepancies. FGF21 mRNA and protein expression in skeletal muscle were also unchanged by CR and ES. Activating transcription factor-4 (ATF4), which is involved in FGF21 expression [38], has been reported to be unaffected by CR [39]. Thus, the response of FGF21 expression in skeletal muscle is expected to be unchanged because CR did not induce any response in the factors upstream of FGF21.

Finally, we measured irisin and FGF21 protein expression in serum. We made the novel discovery that muscle contraction under CR results in a synergistic increase in irisin secretion. A previous study showed that 8 months of CR increased blood irisin levels in mice [35]. Our previous research also showed that acute ES promotes irisin expression in skeletal muscle and plasma [20]. Thus, although each of CR and ES stimulates the secretion of irisin, we found that a synergistic effect occurred when they were applied in combination. Mechanistically, AMPK/FNDC5 cascade signaling may be involved in this synergism. In a previous study, the secretion of irisin induced by exercise was suggested to be due to the AMPK response in muscle and the resulting increase in FNDC5 protein [40]. Another study showed that 8 weeks of CR activated the AMPK signaling pathway in skeletal muscle [41]. Given these previous findings and our own results, it is possible that the synergistic effects of CR and ES on irisin secretion occurred via the permanent increase in AMPK activation levels in myocytes upon 4 weeks of CR, which increased FNDC5 expression in skeletal muscle; acute muscle contraction in turn promoted the cleavage of irisin from FNDC5. FGF21 expression in blood was reduced by CR, despite no change in its mRNA expression levels in skeletal muscle. FGF21 is a cytokine known to be produced by many cell types, including hepatocytes, white and brown adipocytes, skeletal and cardiac myocytes, and pancreatic beta cells [42]. A previous study showed that a low-calorie diet for 7 months reduced FGF21 gene expression in the liver and the plasma FGF21 concentration [43]. It is thus expected that CR reduced FGF21 expression in anatomical locations where it is secreted other than skeletal muscle, resulting in a decrease in the FGF21 concentration in serum. Conversely, some studies have reported that caloric restriction CR increases circulating FGF21 levels [44,45], suggesting that this remains a subject of ongoing debate. Given that circulating FGF21 levels are highly responsive to both acute and chronic environmental and physiological factors [46,47], the decrease in serum FGF21 observed in the present study following CR may be attributable to variables such as pre-sampling fasting, animal age, and housing conditions. Unexpectedly, ES did not increase the expression of FGF21 in blood. A previous study showed that muscle contraction *in vitro* and *in vivo* increased FGF21 gene expression from 15 to 60 min after muscle contraction [48]. As such, our experimental model, in which tissue was collected immediately after ES, likely misses the timepoint of an increase in FGF21. In conclusion, ES under CR promotes irisin secretion more effectively than either intervention alone. We expect that this dynamic variation in irisin is responsible for the synergistic effects of exercise and diet. In future work, we should examine irisin’s role in the adaptation to exercise under CR.

In terms of the limitations of this study, one is that we only measured the relative myokine levels in blood by western blotting. This approach was applied to measure multiple factors from the small blood samples collected from mice, as it can measure multiple factors from a limited sample. However, measurement of absolute concentration by ELISA is also necessary to obtain detailed results.

## Supplementary Information



## Figures and Tables

**Table 1:** Primer sequences for RT-PCR

Gene	Forward primer (5′-3′)	Reverse primer (5′-3′)
*Hprt*	CAGCCCCAAAATGGTTAAGGTT	TCCAACAAAGTCTGGCCTGTAT
*Tbp*	CTGCCACACCAGCTTCTGA	TGCAGCAAATCGCTTGG
*β-actin*	GGCTGTATTCCCCTCCATCG	CCAGTTGGTAACAATGCCATGT
*Fgf21*	CTGCTGGGGGTCACCAAG	CTGCGCCTACCACTGTTCC
*Fndc5*	TTGCTCCAGAATGCAGACCG	TAAACGCGGGGCAGTACCTTC
