# Supplementary figures and images for: Effect of Muscle Contraction Under Caloric Restriction on Irisin and FGF21 Secretion in Mice
Source: Physiol Res. 2025 Dec 1;74(6):969–80. doi: 10.33549/physiolres.935581 (PMC12721820; doi:10.33549/physiolres.935581)

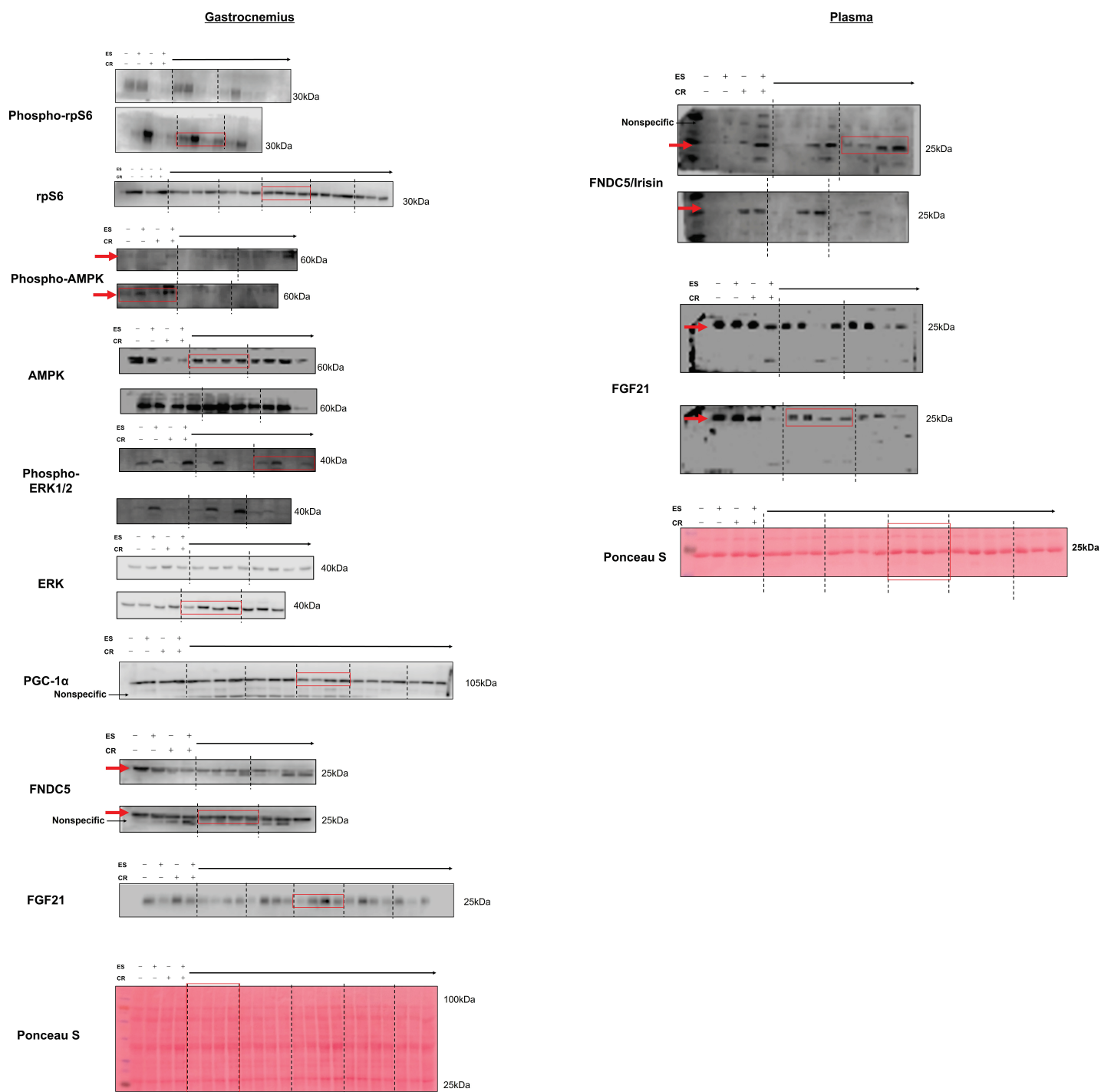

Supplement figure. 1

Supplement: Supplementary file 1 [file Tanimura_Supplement_fig.pdf]
